# Hippocampal subfields in aging: Sex-specific trajectories in structure and hemodynamics

**DOI:** 10.1016/j.neuroimage.2025.121343

**Published:** 2025-06-20

**Authors:** Jiaqi Wen, Chenyang Li, Zhe Sun, Chao Wang, Jiangyang Zhang, Xiaojun Guan, Xiaojun Xu, Thomas Wisniewski, Yulin Ge

**Affiliations:** a Department of Radiology, NYU Grossman School of Medicine, NY, NY 10016, USA; b Department of Radiology, The Second Affiliated Hospital, Zhejiang University School of Medicine 310009, Hangzhou, PR China; c Departments of Neurology, Pathology and Psychiatry, NYU Grossman School of Medicine, NY, NY 10016, USA; d Center for Cognitive Neurology, NYU Grossman School of Medicine, NY, NY 10016, USA

**Keywords:** Aging, Human connectome project, Hippocampal subfields, Hypothalamus, Hemodynamics

## Abstract

Sex differences in hippocampal aging have been increasingly recognized, with females showing greater vulnerability to neurodegeneration, particularly after menopause. However, the underlying neurobiological mechanisms remain unclear, especially at the level of hippocampal subfields. Leveraging high-resolution T1-, T2-weighted, and multi-delay arterial spin labeling MRI from 650 adults in the Human Connectome Project-Aging dataset, we examined sex-specific alterations in hippocampal subfield volume, arterial transit time (ATT), and cerebral blood flow (CBF) across the adult lifespan. All hippocampal subfields showed age-related atrophy and ATT prolongation. An age × sex interaction effect on ATT was observed in CA1 and CA2, indicating that age-related increases in ATT were more pronounced in females than in males in these subfields. Moreover, females exhibited more pronounced hippocampal subfields CBF reductions with aging and atrophy, while males showed relatively preserved CBF, with an increase in subiculum perfusion. Furthermore, CA1 showed the lowest perfusion and the strongest association with atrophy among hippocampal subfields. To investigate the potential impact of menopausal hormonal changes on sex-specific patterns, we explored the hypothalamic structure and hemodynamic alterations during aging and their effects on the hippocampus, given that hypothalamus regulates gonadal hormone secretion through the hypothalamic-pituitary-gonadal axis. We found significant hypothalamic atrophy during aging in both sexes, accompanied by ATT prolongation exclusively in females, which was associated with hippocampal atrophy and impaired hemodynamics. Our study highlights the intricate interplay between hippocampal structure and vascular function, revealing sex- and subfield-specific aging trajectories. These findings provide a normative quantitative imaging reference to age-related neurodegenerative diseases such as Alzheimer’s Disease.

## 1. Introduction

The hippocampus, a complex structure located in the medial temporal lobe, is essential for learning and memory (Maass et al., 2014) and among the primary brain regions affected by Alzheimer’s disease (AD) (Braak and Braak, 1991). While structural magnetic resonance imaging (MRI) has been widely used to assess hippocampal atrophy as an imaging biomarker of neurodegenerative processes (Wolf et al., 2015), it primarily detects irreversible tissue loss and fails to capture early functional alterations that may precede overt atrophy (Jack et al., 2010). Despite growing interest in vascular contributions to age-related cognitive decline and dementia, studies on hippocampal perfusion changes remain limited. Investigating hippocampal hemodynamic changes, in particular at the subfield level, could provide critical insights into early vascular dysfunction, offering potential biomarkers for aging and AD-related neurodegeneration (Kisler et al., 2017; Staffaroni et al., 2019).

Recent histological and neuroimaging studies have highlighted the complexity of the hippocampus, emphasizing the distinct anatomical and functional characteristics of its subfields, including the subiculum, cornu ammonis (CA) fields 1–4, and the dentate gyrus (DG) (Genon et al., 2021; Patel et al., 2020). These subfields contribute differentially to cognitive functions and may exhibit distinct vulnerabilities to aging (Hari et al., 2024). Previous studies have linked hippocampal subfield volume and microstructural changes to cognitive performance in healthy older adults (Malykhin et al., 2024; Wolf et al., 2015). Recent research also supports the feasibility of assessing perfusion changes within hippocampal subfields using advanced neuroimaging techniques (Haast et al., 2024). However, the relationship between hippocampal subfield structural changes and perfusion changes remains poorly understood. Investigating this relationship in large, healthy aging cohorts could provide crucial insights into age-related brain function and cognitive decline.

Additionally, sex differences in hippocampal aging have been reported, with evidence suggesting that females are more susceptible to hippocampal degeneration and associated cognitive decline than males (Torromino et al., 2021). A key factor may be hormonal fluctuations, particularly estrogen decline during menopause, which has been implicated in accelerating neurodegenerative processes. Estrogen is known to exert neuroprotective effects by promoting synaptic plasticity, enhancing cerebral blood flow (CBF), and mitigating oxidative stress (Martínez-Martos et al., 2024), and its depletion may exacerbate hippocampal aging. Understanding sex-specific patterns in structural and perfusion alterations through large-scale and data-driven analysis could shed light on the sex-specific mechanisms of brain aging and inform targeted interventions for cognitive decline.

Arterial spin labeling (ASL) perfusion imaging is a non-invasive MRI technique that quantifies regional CBF, offering critical insights into cerebral perfusion dynamics (Detre et al., 1992; Thropp et al., 2024). A recent study using ultra-high-field (7T) ASL revealed that the CA1 subfield exhibits the lowest perfusion levels within the hippocampus in young adults (Haast et al., 2024). However, applying single post-labeling delay (PLD) ASL in aging populations is challenging due to age-related vascular changes, such as prolonged arterial transit time (ATT) caused by increased vessel tortuosity (Sun et al., 2022; Zhang et al., 2022). If unaccounted for, delayed ATT can compromise the accuracy of CBF measurements, potentially leading to misinterpretations of normal aging versus pathological alterations. Multi-delay pseudo--continuous ASL (pCASL), which acquires perfusion data at multiple PLDs, enables a more precise characterization of arterial inflow dynamics and enhances CBF quantification across different vascular territories (Woods et al., 2024).

This study utilized Human Connectome Project-Aging (HCP-Aging) (Bookheimer et al., 2019), including high-resolution T1- and T2-weighted MRI, multiple PLD pCASL, and cognitive assessments from a large adult lifespan cohort of healthy individuals. To enhance hippocampal subfield characterization, we applied the HippUnfold approach (DeKraker et al., 2022), which improves inter-subject alignment and subfield parcellation while preserving topological integrity. We investigated sex-specific structural and hemodynamic alterations in hippocampal subfields and explored the impact of menopausal hormonal changes by assessing age-related hypothalamic changes, given the hypothalamus’s role in regulating gonadal hormones via the hypothalamic-pituitary-gonadal (HPG) axis (Xu et al., 2025). Findings from this large and well-characterized healthy cohort may offer normative references for atypical hippocampal aging and inform early detection and intervention strategies for AD and AD-related dementia.

## 2. Materials and methods

### 2.1. Participants in the HCP-Aging dataset

The participants in the HCP-Aging dataset are healthy volunteers, representing normative aging within their respective age groups. They have no identified pathological causes of cognitive decline, such as stroke, tumors, or clinically diagnosed dementia (Harms et al., 2018). All participants provided written informed consent, and the study received approval from the Institutional Review Board (IRB) as part of a cross-sectional research initiative. Participants over the age of 90 were excluded due to potential inaccuracies in reported age data. Detailed information on the inclusion/exclusion criteria for the HCP-Aging dataset has been documented in previous studies (Bookheimer et al., 2019).

Finally, this study analyzed data from 650 participants who completed the full imaging protocol, which included high-resolution T1-weighted, T2-weighted, and multiple PLD pCASL scans. Cognitive function was assessed using the Total Cognition Composite Score, which integrates both the Fluid Cognition Composite and the Crystallized Cognition Composite scores (Akshoomoff et al., 2014; Heaton et al., 2014). The Fluid Cognition Composite comprises tasks that evaluate cognitive domains known to be sensitive to aging and neurological disorders (e.g., processing speed, memory, executive functioning), while the Crystallized Cognition Composite includes tests that reflect premorbid intellectual functioning (e.g., oral reading recognition) (Scott et al., 2019). To examine the influence of menopause-related hormonal changes, female participants were stratified into three age groups: 36–45 years (pre-menopausal), 45–55 years (peri-menopausal), and 55–90 years (post-menopausal). This approach accounted for individual variability in the onset of menopause or peri-menopausal period (Davis et al., 2015; Ebong et al., 2022). For comparison, male participants were grouped using the same age ranges.

### 2.2. MRI data acquisition

The MRI data in HCP-Aging participants were acquired using a Siemens Prisma 3 Tesla scanner with a 32-channel head coil (Harms et al., 2018). A T1-weighted anatomical scan was obtained using a magnetization-prepared rapid acquisition gradient echo (MPRAGE) sequence with the following parameters: voxel size = 0.8 × 0.8 × 0.8 mm^3^, echo times (TE) = 1.8/3.6/5.4/7.2 ms, repetition time/inversion time (TR/TI) = 2500/1000 ms, flip angle (FA) = 8°, and number of echoes = 4. T2-weighted images were acquired using a variable-flip-angle turbo spin echo (TSE) sequence (Siemens SPACE) with the following parameters: TR/TE = 3200/564 ms, turbo factor = 314, and voxel size = 0.8 × 0.8 × 0.8 mm^3^. pCASL data were acquired without background suppression as previously described (Li et al., 2015; Xiufeng Li et al., 2018). The labeling duration was set to 1500 ms, with five PLDs of 200 ms (control/label pairs = 6), 700 ms (pairs = 6), 1200 ms (pairs = 6), 1700 ms (pairs = 10), and 2200 ms (pairs = 15). Signal readout was conducted using multi-band 2D gradient echo-planar imaging (EPI) with the following parameters: TR = 3580 ms, TE = 19 ms, partial Fourier = 6/8, simultaneous multi-slice acquisition (SMS) acceleration factor = 6, total slices = 60, voxel size = 2.5 × 2.5 × 2.5 mm^3^, and a total acquisition time of approximately 5.5 min. Two equilibrium magnetization (M_0_) scans were acquired at the end of the session to calibrate cerebral blood flow (CBF) quantification. For field mapping and distortion correction, a pair of spin-echo EPI scans with opposite phase encoding directions were acquired, with a spatial resolution of 2.5 mm isotropic and TR/TE values of 8000/40 ms.

### 2.3. ASL data preprocessing

**Fig. 1.**
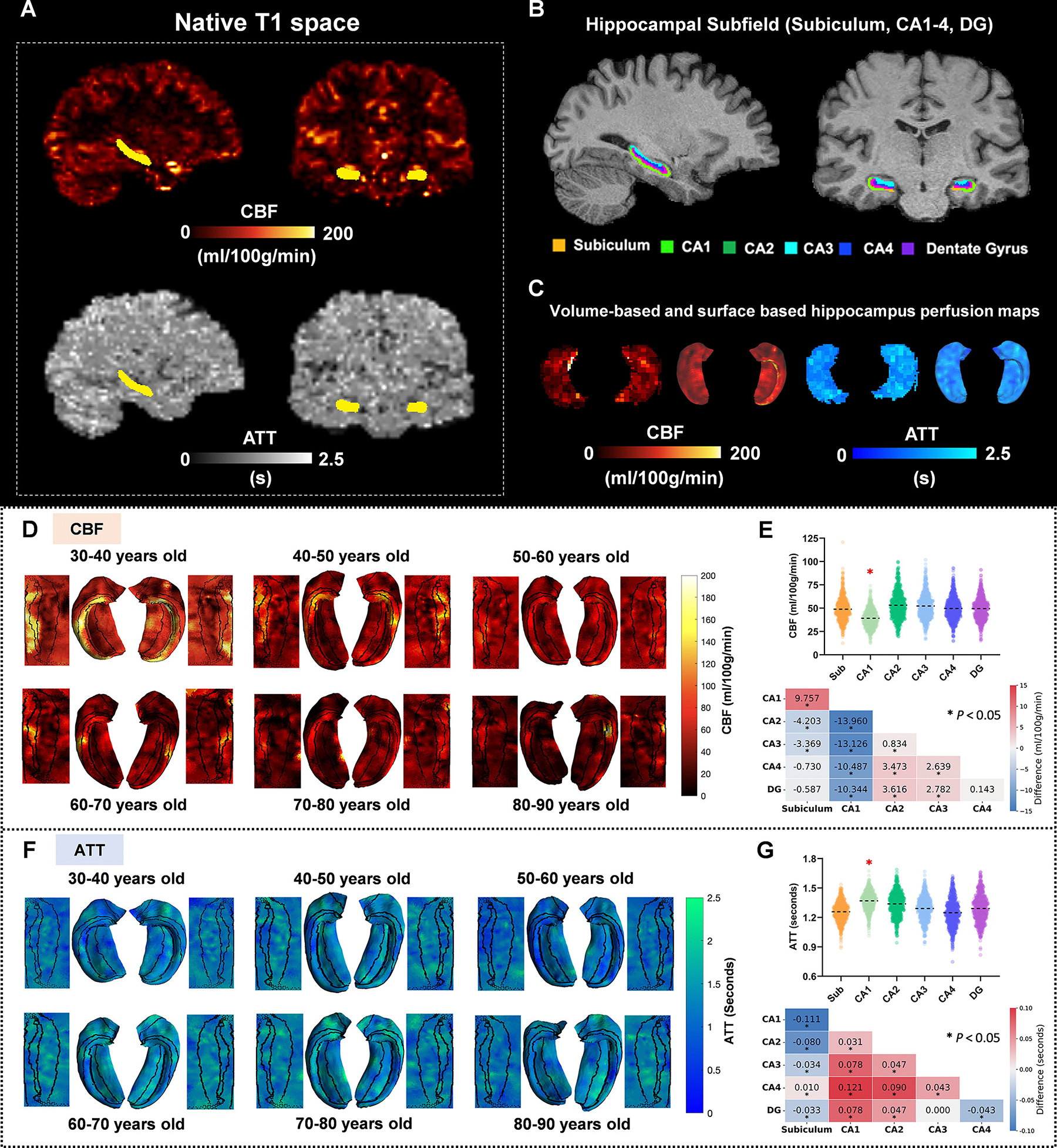
(**A**) Representative cerebral blood flow (CBF) and arterial transit time (ATT) maps in native T1 space, with whole hippocampus masks overlaid. (**B**) Representative hippocampus subfield masks on T1-MPRAGE data. (**C**) Volume-based and surface-based projection of the hippocampus CBF and ATT to create spatial perfusion measurement maps. Representative CBF (**D**) and ATT (**F**) maps in the left and right hippocampus across the adult lifespan, with data in each decade. ATT and CBF were spatially projected onto both hippocampal and hippounfolded structures for the extraction of CBF and ATT values within each subfield. Among the hippocampal subfields, CA1 showed the lowest CBF (**E**) and longest ATT (**G**) (*P* < 0.001). The dashed line (top figure of E and G) represents the average value. Pairwise comparisons of subfield averages are shown in heatmaps (bottom figure of E and G), with statistically significant *p*-values (< 0.05) after false discovery rate (FDR) correction marked by asterisks (*).

The multiple PLD pCASL data was preprocessed using the hcpasl minimal processing pipeline (McConnell et al., 2022) (https://github.com/physimals/hcp-asl) to generate CBF and ATT maps (Fig. 1A). The initial phase involved the correction of the label and control ASL images to address a linear intensity variation, referred to as the banding effect, within each 10-slice band, attributed to magnetization transfer (MT) effects. Additionally, the data underwent correction for slice-wise SMS-related saturation-recovery intensity variation by fitting the saturation recovery curve. Motion correction was implemented to enhance saturation recovery fitting and align the de-banded ASL data. Susceptibility distortion correction was carried out using TOPUP with phase encoding-reversed spin-echo images (Andersson et al., 2003). Subsequently, post-alignment with the pre-processed T1-weighted image, perfusion analysis was performed on the ASL data while preserving the original 2.5 mm isotropic ASL voxel grid. Following pre-processing, a general linear model was employed for label-control subtraction, aiding in the rescaling of the perfusion-weighted signal and its separation from through-slice motion variations (Suzuki et al., 2019). Computation of CBF and ATT was executed using the Bayesian Inference for Arterial Spin Labeling (BASIL) toolkit within FSL (version 6.0.5, Oxford, UK), with partial volume correction (Chappell et al., 2011). This approach facilitated fitting a two-compartment exchange model to independently estimate tissue and macrovascular contributions to the perfusion signal (Parkes and Tofts, 2002).

### 2.4. Hypothalamus segmentation

T1-weighted images were first preprocessed using the ‘recon-all’ command in the FreeSurfer 6.0 pipeline (http://surfer.nmr.mgh.harvard.edu/). The ‘recon-all’ procedure executed the full reconstruction pipeline, including cortical and subcortical segmentation as well as volume measurement (Fischl et al., 2002). Additionally, the estimated total intracranial volume (eTIV), comprising gray matter, white matter, and cerebrospinal fluid, was calculated. The preprocessing pipeline consisted of several key steps: non-uniform intensity normalization, Talairach transformation, skull stripping, subcortical segmentation, intensity normalization (applied to the skull-stripped brain volume), white matter segmentation, tessellation of the gray-white matter boundary, automatic topology correction, generation of final pial and white matter surfaces, and cortical parcellation. Following these steps, the hypothalamus was automatically segmented, and its volume was simultaneously estimated using the subcortical processing stream available in FreeSurfer 7.2 (Billot et al., 2020).

### 2.5. Hippocampal subfields segmentation

Hippocampal subfields (subiculum, CA1–4, and DG) were segmented using the HippUnfold application (v1.2.0 release at https://zenodo.org/record/7063098) (DeKraker et al., 2022) (Fig. 1B) with T1- and T2-weighted images. The workflow included: (1) MRI pre-processing (non-uniformity correction, resampling to 0.3 mm isotropic subvolumes, registration, and cropping); (2) Automatic segmentation via deep convolutional neural network U-net (nnU-net) (Isensee et al., 2021), followed by post-processing with fluid label-label registration to a high-resolution average template; (3) Imposing hippocampal coordinates across the anterior-posterior, proximal-distal, and inner-outer dimensions of hippocampal gray matter by solving Laplace’s equation, with an equivolume solution applied for laminar coordinates; (4) Generating transformations between native and unfolded spaces using scattered interpolation on the hippocampal coordinates; (5) Applying these transformations to generate hippocampal surfaces meshes, with subfield labels mapped from the histological BigBrain atlas of the hippocampus to individual space; and (6) Generating high-resolution volumetric subfield segmentation by applying the same transformations and coordinate representations.

### 2.6. Hippocampal subfields and hypothalamus hemodynamics quantification

The low-resolution T1-weighted images of the ASL grid generated by the ASL pipeline were registered to the native high-resolution T1-weighted images, and then the transformation matrix was applied to CBF and ATT maps to obtain perfusion maps in the individual T1w space. CBF and ATT in hippocampal subfields were unfolded and projected into the hippocampal unfolded space to acquire subfield-specific values. Connectome Workbench was used to realize volume-to-surface mapping and visualization (Fig. 1D, F) (Marcus et al., 2011). To mitigate partial volume effects, the hypothalamus mask was eroded by one voxel, and CBF and ATT values were then extracted for analysis.

### 2.7. Statistical analysis

#### 2.7.1. Comparisons of clinical and imaging data across age groups

Data normality was assessed using the Kolmogorov-Smirnov test. The general linear model with post hoc analysis was employed to compare hippocampal subfield and hypothalamus volumes, ATT, CBF, and total cognition composite scores across age groups in males and females separately. Effect sizes for group comparisons were reported using partial η^2^. Paired *t*-tests examined hemodynamic differences among hippocampal subfields. To control for false positives due to multiple comparisons, p-values were adjusted using the false discovery rate (FDR) correction.

#### 2.7.2. Correlation analysis of age, volume, hemodynamics, and cognition

To determine the most appropriate model for analyzing age-related changes, an extra sum-of-squares F test was conducted to determine whether the second-order polynomial (quadratic) parameter (B2) significantly differed from the null hypothesis. A quadratic model ($Y=B0+B1\times Agc+B2\times Age^{2}$) was selected if the *p*-value was <0.05, and the coefficient of determination (R^2^) was reported as the effect size. Otherwise, a linear model was used to assess associations among age, cognition, and imaging measures of the hippocampal subfields and hypothalamus. For normally distributed variables, Pearson’s correlation analysis was applied, with the correlation coefficient (r) serving as the effect size. For non-normally distributed variables, Spearman’s rank correlation was used, and the correlation coefficient (ρ) was reported as the effect size. P-values were adjusted using the FDR correction to control for false positives.

#### 2.7.3. Age × sex interaction analysis

To evaluate whether the slopes of age-related linear trajectories in hemodynamic measures (e.g., CBF and ATT) differ between males and females, a linear regression model including an age × sex interaction term was applied to each hippocampal subfield’s ATT and CBF. The model was specified as: Outcome=β0+β1(Age)+β2(Sec)+β3(Age×Sex). A significant interaction term (β3, *p* < 0.05) indicated the sex-specific differences in the slopes of age-related changes in CBF and ATT.

In the above volumetric analyses, all brain region volumes were normalized by eTIV to account for individual head size variations. Statistical analyses were conducted using SPSS and Prism, with a significance threshold of *p* < 0.05 after FDR correction.

## 3. Results

### 3.1. Demographic and imaging information of participants

From large-scale neuroimaging datasets of HCP-Aging, the 650 participants (mean age: 60, range: 36–90) included 358 females and 292 males. Among females, 76 were aged 36–45, 81 were aged 45–55, and 201 were aged 55–90. Among males, 58 were aged 36–45, 59 were aged 45–55, and 175 were aged 55–90. In females, the total cognition composite score was significantly lower in the 55–90 age group compared to both the 45–55 (*P*_female_ = 0.003) and 36–45 (*P*_female_ < 0.001) age groups. In males, a significant reduction in cognitive performance was observed only when comparing the 55–90 and 36–45 age groups (*P*_male_ = 0.003). Regarding imaging results, all hippocampal subfields exhibited significantly reduced volumes and prolonged ATT in the 55–90 age group compared to both the 36–45 and 45–55 age groups in both sexes (*P*_female_ ≤ 0.032; *P*_male_ ≤ 0.038). In females, CBF was significantly decreased in the CA1–4 and DG subfields in the 55–90 age group compared to the 45–55 age group (*P*_female_ ≤ 0.028), whereas in males, a significant CBF reduction was observed only in CA2 (*P*_male_ = 0.025). When compared to the 36–45 age group, CBF was reduced in CA1–3 and DG in females (*P*_female_ ≤ 0.048), and in CA1–3 in males (*P*_male_ ≤ 0.042). The age-related effect sizes of hippocampal subfield CBF were greater in females than in males. In both sexes, hippocampal subfield volumes showed medium to large age-related effects; ATT exhibited large age-related effects, while CBF demonstrated small to medium effects. Detailed effect sizes are provided in Table 1. No significant differences were found between the 36–45 and 45–55 groups in any hippocampal subfield measurements. All reported p-values were corrected for multiple comparisons. Detailed demographic and imaging information across age groups in females and males is provided in Table 1.

### 3.2. Hemodynamics in hippocampal subfields

Fig. 1A shows representative CBF and ATT maps, registered to the native T1 space and corrected for partial volume effects using the BASIL toolkit in FSL (Chappell et al., 2011). To enhance interpretability, these maps were projected onto the unfolded hippocampal surface, where the subfields—subiculum, CA1, CA2, CA3, CA4, and DG subfields were arranged from inside to outside (Fig. 1D, F). Surface-based mapping and unfolding of the pCASL data revealed a spatially heterogeneous perfusion pattern within the hippocampus. The specific group-level ATT and CBF values were presented in Table 1. Particularly, CA1 showed the lowest CBF (average of 39.178 ml/100 g/min) and the longest ATT (average of 1.368 s) among hippocampal subfields in all populations, as indicated by pairwise comparisons (*P* < 0.001, Fig. 1E, G). These findings are consistent with those reported by Haast et al. (Haast et al., 2024). Furthermore, our study characterized hippocampal subfield hemodynamics due to vascular aging, revealing a progressive reduction in CBF and a prolongation of ATT. Representative CBF and ATT images across different age groups visually illustrated these trends (Fig. 1D, F).

### 3.3. Sex-Specific structural and hemodynamic trajectories in hippocampal subfields

**Fig. 2.**
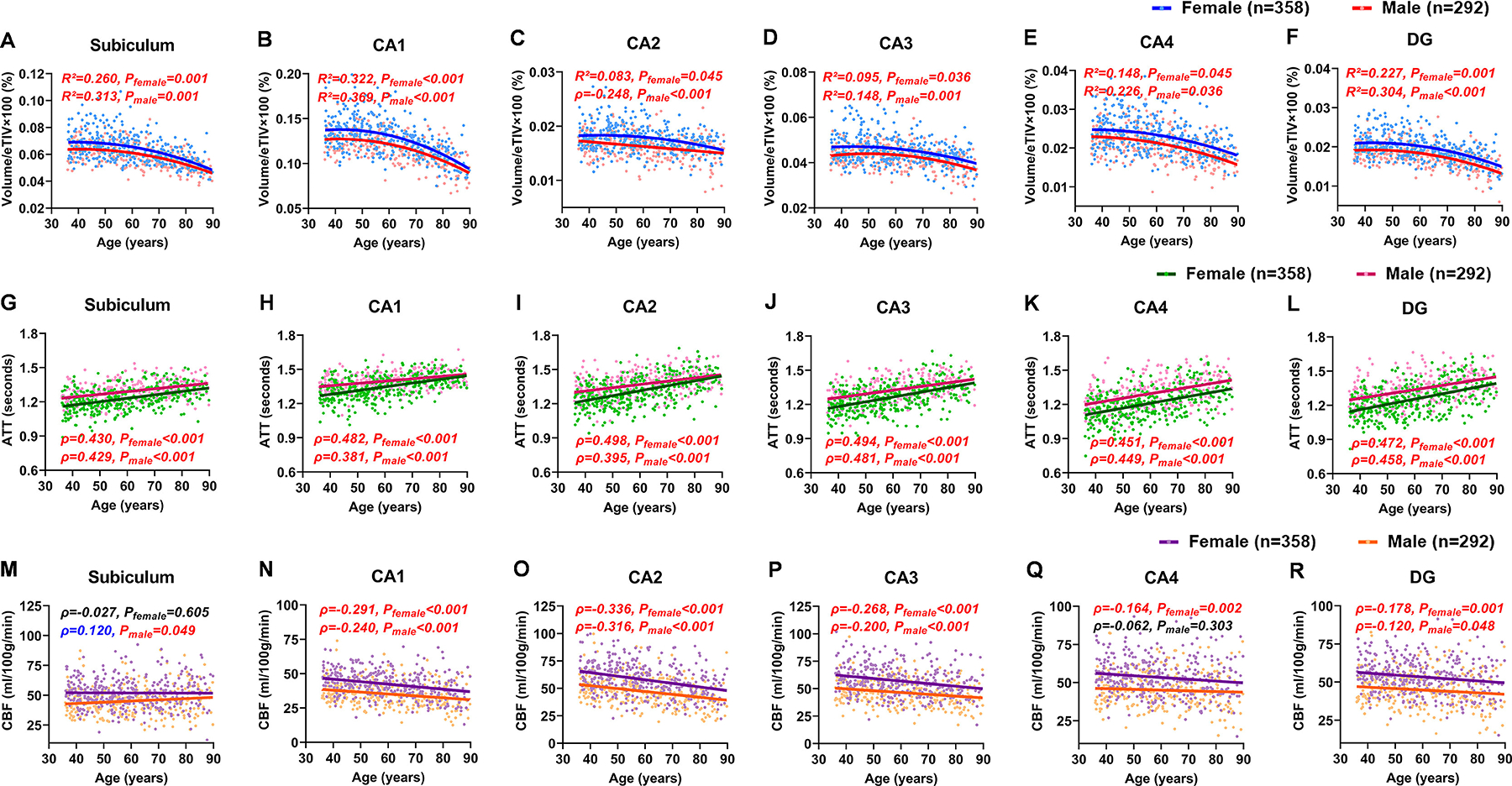
Sex-specific trajectories of hippocampal subfield volumes, cerebral blood flow (CBF), and arterial transit time (ATT) with age. (**A–F**) The volumes of all hippocampal subfields exhibited quadratic nonlinear correlations with age, except for CA2 vol in males, which showed a linear decline with age. (**G–L**) ATT was positively correlated with age across all hippocampal subfields in both sexes. (**M–R**) CBF was negatively correlated with age in the CA1–3 and DG in both sexes, as well as in the CA4 in females. In males, subiculum CBF was positively correlated with age. For linear correlations, Pearson’s correlation coefficients (r) were reported for normally distributed variables, and Spearman’s rank correlation coefficients (ρ) for non-normally distributed variables. For nonlinear associations modeled with quadratic regression, the coefficient of determination (R^2^) was reported. *P*_female_ and *P*_male_ refer to *p*-values derived from sex-stratified correlation analyses for females and males, respectively. Statistically significant correlations (*p* < 0.05, FDR-corrected) are highlighted in red.

The volumes of all hippocampal subfields exhibited quadratic nonlinear negative correlations with age (*P*_female_ < 0.05, *P*_male_ < 0.05, Fig. 2A–F), except for CA2 in males, which showed a linear negative correlation with age (ρ = −0.248, *P*_male_ < 0.001, Fig. 2C). These quadratic relationships indicate accelerated atrophy of hippocampal subfields in the late-life. The fitted quadratic regression equations for age-related volume changes in each hippocampal subfield for males and females are provided in the Supplementary Material 1.

The optimal capture of noninvasive blood perfusion measurements, including hippocampal ATT, is achieved using multiple PLD pCASL, an ASL sequence recommended by the white paper for clinical use (Alsop et al., 2015). ATT of all hippocampal subfields demonstrated linear positive correlations with age (*P*_female_ < 0.001, *P*_male_ < 0.001, Fig. 2G–L). Additionally, interaction analysis revealed a significant age × sex interaction effect on ATT in CA1 (β3 = −0.429, *P* = 0.003) and CA2 (β3 = −0.384, *P* = 0.008), indicating that the age-related increase in ATT was more pronounced in females than in males in these subfields (see Supplementary Material 2 for detailed results). CBF also exhibited sex-specific trajectories with age (Fig. 2M–R). In females, the CBF of all hippocampal subfields, except for the subiculum (*P*_female_ = 0.605), showed negative linear correlations with age (*P*_female_ ≤ 0.002). In males, only the CBF of CA1–3 and DG showed negative linear correlations with age (*P*_male_ ≤ 0.048), whereas the CBF of the subiculum was positively correlated with age (ρ = 0.120, *P*_male_ = 0.049).

### 3.4. Hippocampal subfield volume-hemodynamics relationship

**Fig. 3.**
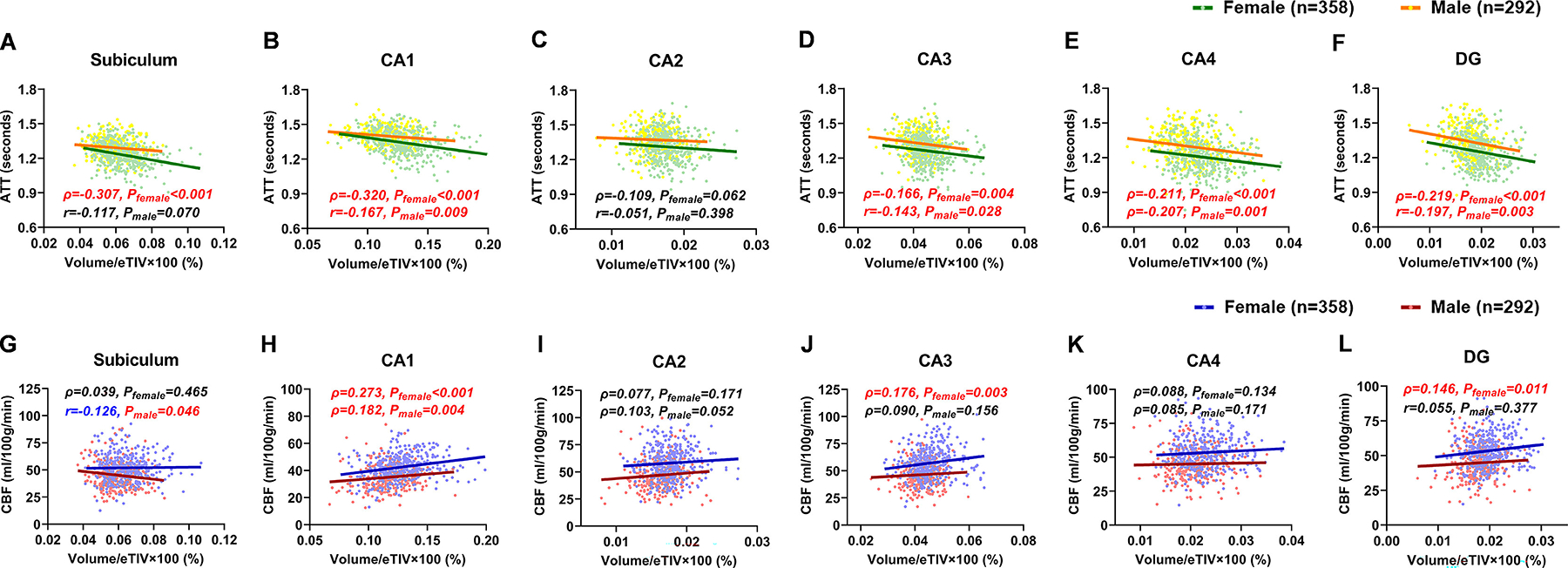
Sex-specific correlations between hippocampus subfield volumes and hemodynamics. (**A–F**) Scatter plots depicting correlations between hippocampus subfield volumes and arterial transit time (ATT). The volumes of CA1, CA3, CA4, and DG in both sexes, as well as the subiculum in females, showed negative correlations with ATT. (**G–L**) Scatter plots illustrating correlations between hippocampal subfield volumes and cerebral blood flow (CBF). Positive correlations between hippocampal subfield volumes and CBF were observed only in CA1 for both sexes, as well as in CA3 and DG for females. Notably, subiculum volume negatively correlated with CBF in males. Pearson’s correlation coefficients (r) were reported for normally distributed variables, and Spearman’s rank correlation coefficients (ρ) for non-normally distributed variables. *P*_female_ and *P*_male_ refer to *p*-values derived from sex-stratified correlation analyses for females and males, respectively. Statistically significant correlations (*p* < 0.05, FDR-corrected) are highlighted in red.

In both sexes, ATT of CA1, CA3, CA4, and DG exhibited negative correlations with volumes (*P*_female_ ≤ 0.004, *P*_male_ ≤ 0.028, Fig. 3B, D–F), whereas ATT of CA2 showed no correlation with volume (*P*_female_ = 0.062, *P*_male_ = 0.398, Fig. 3C). ATT of subiculum was negatively correlated with age only in females (*P*_female_ < 0.001, *P*_male_ = 0.070, Fig. 3A).

In both sexes, only CBF of CA1 exhibited a positive correlation with volume (*P*_female_ < 0.001, *P*_male_ = 0.004, Fig. 3H), while CBF of CA2 and CA4 showed no correlation with volume (CA2: *P*_female_ = 0.171, *P*_male_ = 0.052, Fig. 3I; CA4: *P*_female_ = 0.134, *P*_male_ = 0.171, Fig. 3K). CBF of CA3 and DG were positively correlated with volume only in females (CA3: *P*_female_ = 0.003, *P*_male_ = 0.156, Fig. 3J; DG: *P*_female_ = 0.011, *P*_male_ = 0.377, Fig. 3L). Notably, subiculum volume demonstrated a negative correlation with CBF in males (*r* = −0.126, *P*_male_ = 0.046, Fig. 3G).

### 3.5. Linking cognition to hippocampal subfields volumes and hemodynamics

**Fig. 4.**
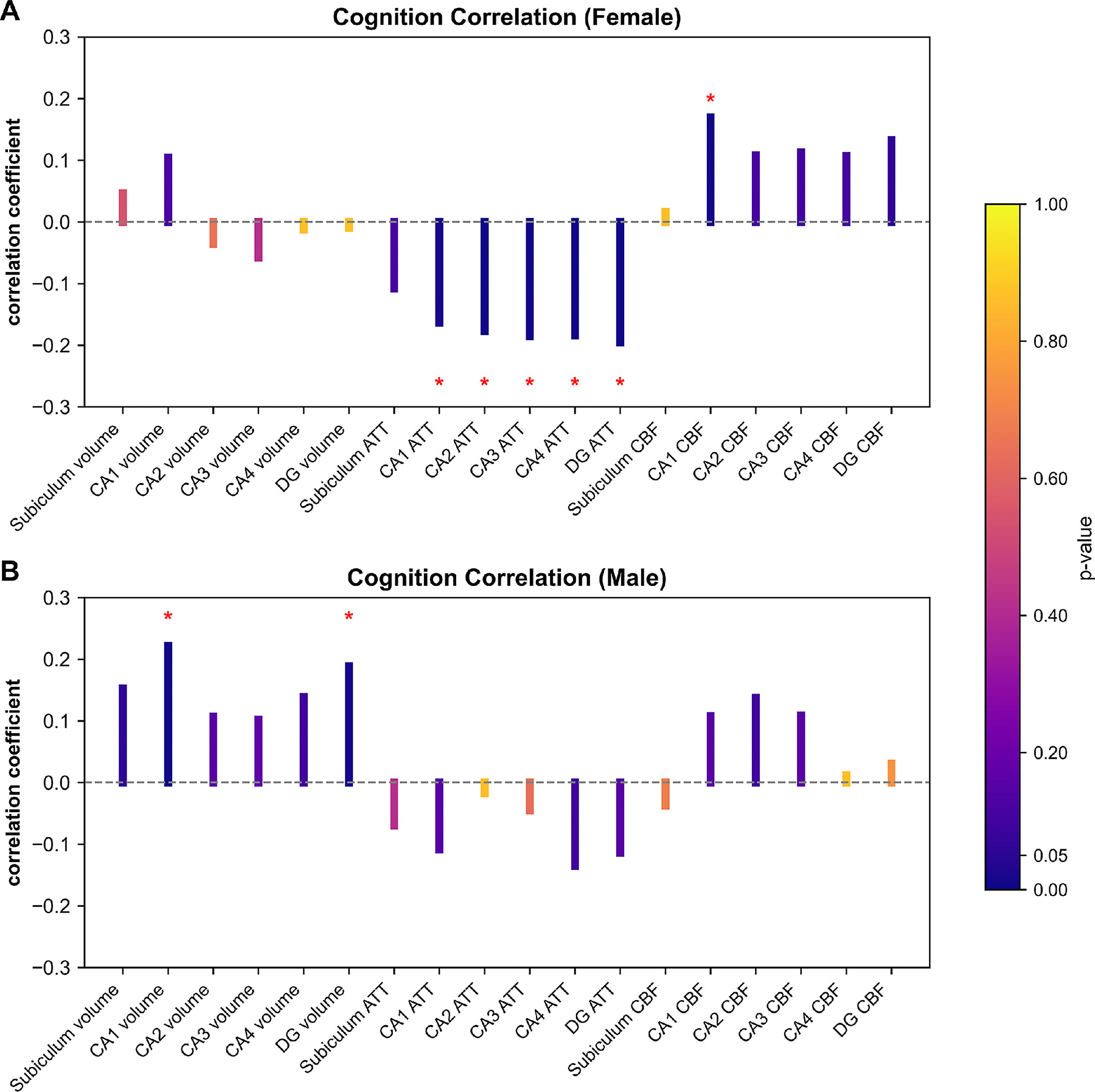
Associations between total cognition composite score and hippocampal subfield volumes, arterial transit time (ATT), and cerebral blood flow (CBF). (**A**) In females, ATT in CA1–4 and DG showed negative correlations with the total cognition composite score; CBF in CA1 exhibited positive correlations. (**B**) In males, the volumes of CA1 and DG were positively correlated with the total cognition composite score. Statistically significant *p*-values (< 0.05) after false discovery rate (FDR) correction are marked by asterisks (*).

We further investigated how age-related hippocampal subfields changes affect a comprehensive battery of cognitive assessments, derived from the NIH Toolbox Cognitive Battery (Akshoomoff et al., 2014). The correlation maps presented in Fig. 4 illustrate the associations between cognitive function and hippocampal subfield volumes as well as hemodynamic properties. Specifically, in females, ATT of CA1–4 and DG was negatively correlated with the total cognition composite score (CA1: ρ = −0.163, *P*_female_ = 0.016; CA2: ρ = −0.177, *P*_female_ = 0.011; CA3: ρ = −0.185, *P*_female_ = 0.009; CA4: ρ = −0.184, *P*_female_ = 0.009; DG: ρ = −0.195, *P*_female_ = 0.009); CBF of CA1 showed a positive correlation (ρ = 0.170, *P*_female_ = 0.014). In males, the volumes of CA1 and DG were positively correlated with the total cognition composite score (CA1: *r* = 0.222, *P*_male_ = 0.009; DG: *r* = 0.189, *P*_male_ = 0.016). These results revealed that in aging males, hippocampal volume was predominantly correlated with cognitive function, whereas in aging females, hippocampal hemodynamics played a more significant role in cognitive performance. However, after adjusting for age, the correlations between cognition and hippocampal subfield measures were no longer significant in either sex, suggesting that the observed sex-specific correlations might be age-dependent.

### 3.6. Hypothalamic and hippocampal structure-hemodynamics links in aging

**Fig. 5.**
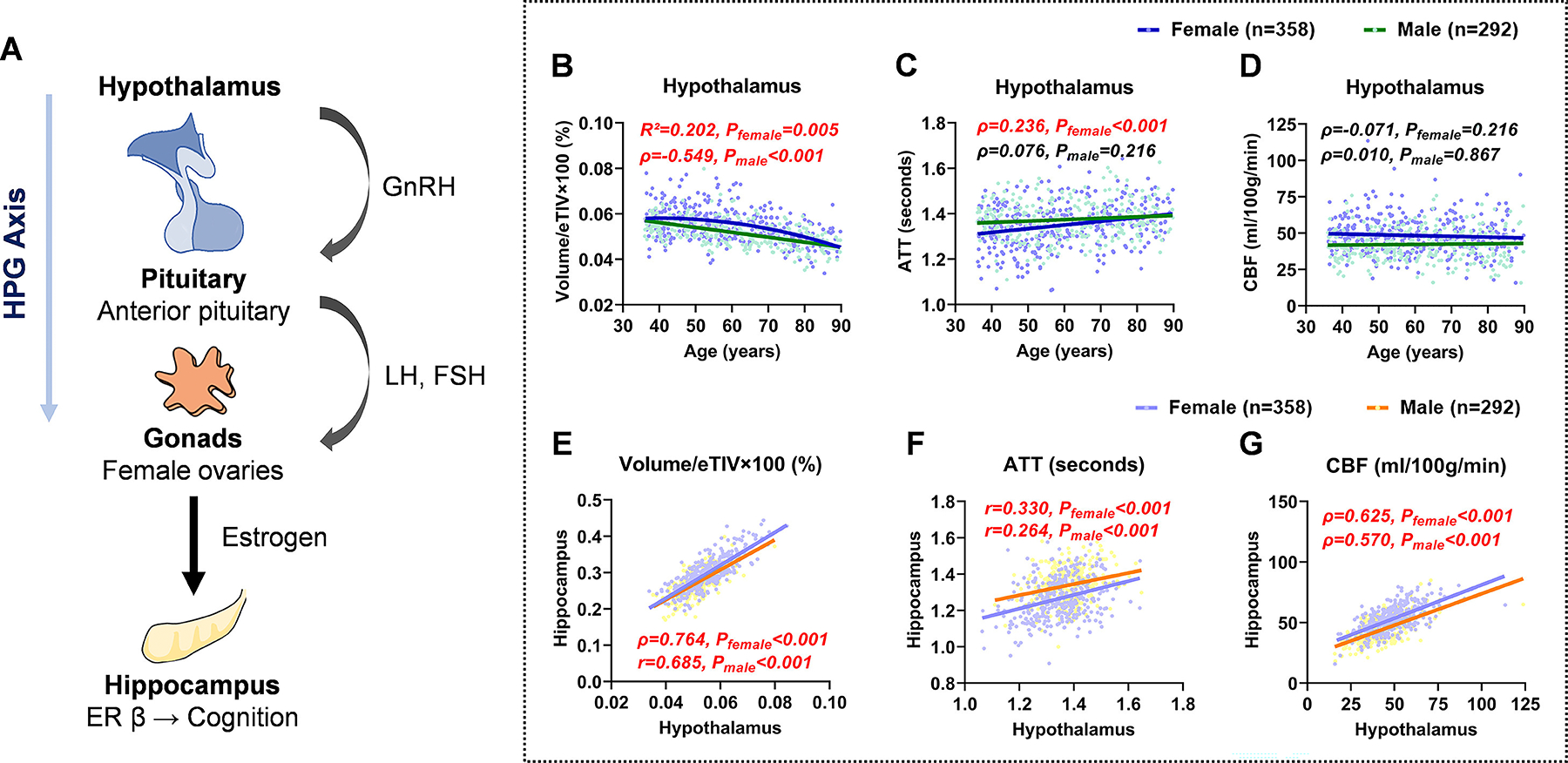
Associations between hypothalamic and hippocampal structure and hemodynamics. (**A**) Schematic representation of the hypothalamic-pituitary-gonadal (HPG) axis and its connection to the hippocampus. The hypothalamus regulates gonadal hormone secretion by releasing gonadotropin-releasing hormone (GnRH), which stimulates the anterior pituitary to secrete luteinizing hormone (LH) and follicle-stimulating hormone (FSH). In females, LH and FSH further promote ovarian secretion of estrogen, which modulates hippocampal function via estrogen receptor β (ER β), influencing cognition. (**B-D**) Associations between hypothalamic volume, ATT, CBF, and age. Hypothalamic atrophy was observed with aging in both sexes, whereas ATT prolongation was specific to females. (**E-G**) Positive correlations were observed among hypothalamic and hippocampal volume, ATT, and CBF. For linear correlations, Pearson’s correlation coefficients (r) were reported for normally distributed variables, and Spearman’s rank correlation coefficients (ρ) for non-normally distributed variables. For nonlinear associations modeled with quadratic regression, the coefficient of determination (R^2^) was reported. *P*_female_ and *P*_male_ refer to *p*-values derived from sex-stratified correlation analyses for females and males, respectively. Statistically significant correlations (*p* < 0.05, FDR-corrected) are highlighted in red.

To investigate the potential impact of menopausal hormonal changes on sex-specific patterns, we explored the age-related hypothalamic structure and hemodynamic alterations and their effects on the hippocampus, given that the hypothalamus regulates gonadal hormone secretion through the HPG axis (Fig. 5A). As shown in Table 1, hypothalamic volume significantly decreased in both sexes in the 55–90 age group compared to the 36–45 and 45–55 age groups (*P*_female_ < 0.001, *P*_male_ < 0.001). ATT in the hypothalamus increased only in females (*P*_female_ < 0.001 for 45–55 vs. 55–90 age group, *P*_female_ = 0.006 for 36–45 vs. 55–90 age group). No significant differences in hypothalamic CBF were observed among age groups in either sex. Correlation analysis further confirmed these age-related hypothalamic alterations (Fig. 5B–D). In females, hypothalamus volume exhibited a quadratic negative correlation with age (*Hypothalamus volume* = 0.050+ 4.171 × 10^−4^ × *Age* − 5.202 × 10^−6^ × *Age*^2^, *P*_female_ = 0.005), whereas in males, it followed a linear negative correlation (ρ = −0.549, *P*_male_ < 0.001). ATT increased significantly with age in females, but remained unchanged in males (ρ = 0.236, *P*_female_ < 0.001; ρ = 0.076, *P*_male_ = 0.216). Hypothalamic CBF was not significantly correlated with age in both sexes (ρ = −0.071, *P*_female_ = 0.216; ρ = 0.010, *P*_male_ = 0.867). Importantly, hypothalamic volume (ρ = 0.764, *P*_female_ < 0.001; *r* = 0.685, *P*_male_ < 0.001), ATT (*r* = 0.330, *P*_female_ < 0.001; *r* = 0.264, *P*_male_ < 0.001), and CBF (ρ = 0.625, *P*_female_ < 0.001; ρ = 0.570, *P*_male_ < 0.001) were positively correlated with their respective hippocampal metrics in both sexes (Fig. 5E–G), indicating synchronized structural and hemodynamic integrity between these two regions.

## 4. Discussion

In this study, by leveraging high-resolution T1- and T2-weighted MRI and multiple-PLD pCASL data from 650 healthy individuals in the HCP-Aging dataset, we presented age- and sex-specific structural and hemodynamic alterations in hippocampal subfields in mid- and late-life of a large-scale healthy population. The key findings are as follows: (1) Perfusion in the hippocampal subfields appeared more vulnerable to aging and atrophy in females than in males, potentially linked to hypothalamic structural and hemodynamic impairment following menopause, whereas males exhibited an age-related increase in subiculum CBF. (2) CA1 hemodynamics, with the lowest CBF and longest ATT, showed the strongest association with age-related atrophy among the hippocampal subfields in both sexes. (3) Compared to CBF, ATT offered important complementary insights into age- and sex-related vascular function changes in the hippocampal subfields. Therefore, ATT, a unique perfusion measure derived from multiple PLD pCASL, is essential for gaining additional insights into age-related vascular function alterations.

### 4.1. Sex differences in hippocampal subfields hemodynamics during aging

Hemodynamics of the hippocampal subfields in females appeared more susceptible to aging and atrophy than in males. In males, increased CBF in the subiculum may compensate for subfield atrophy and reduced CBF in other subfields. Estrogen, via estrogen receptor beta (ERβ), highly expressed in the hippocampus, supports hippocampal plasticity through the long-term potentiation of synaptic transmission (Baek et al., 2024; Liu et al., 2008). However, menopause-induced estrogen loss impairs this function, increasing memory decline and AD risk in females (Camon et al., 2024). In contrast, males may compensate through the subiculum’s unique vascular architecture, where collateral branches from the P2 segment of the posterior cerebral artery with a relatively larger diameter help maintain perfusion (Henri et al., 2013), highlighting its role in preserving hippocampal function with aging.

To further investigate the impact of estrogen decline on hippocampal structure and hemodynamics in aging females, we examined age-related changes in the hypothalamus. The hypothalamus regulates gonadal hormone secretion by releasing gonadotropin-releasing hormone, which stimulates luteinizing and follicle-stimulating hormones (Xu et al., 2025). In females, estrogen modulates hippocampal function through ERβ, influencing cognition (Liu et al., 2008) (Fig. 5A). While both sexes showed hypothalamic atrophy with age, only females exhibited prolonged ATT, linked to hippocampal atrophy and impaired hemodynamics. The hypothalamus and hippocampus have distinct vascular supplies, with the hippocampus mainly supplied by branches of the posterior cerebral artery (Henri et al., 2013) and the hypothalamus by perforating arteries from the circle of Willis (Lechan and Toni, 2000), but both structures are influenced by systemic factors such as estrogen deficiency and vascular aging. Therefore, changes in hypothalamic hemodynamics may reflect post-menopausal alterations that also affect the hippocampus. Additionally, hippocampal volume correlated with cognition in aging males, whereas hippocampal hemodynamics played a larger role in aging female cognition. Given the pronounced age-related decline in hippocampal hemodynamics observed in females, hormone replacement or vascular modulation therapies may help mitigate cognitive decline in females during typical and atypical aging.

### 4.2. Selective vulnerability of CA1 during aging

Among hippocampal subfields, CA1 exhibited the lowest hemodynamic levels and the strongest correlation with age-related atrophy. A 7T MRI study suggested that CA1’s reduced perfusion may stem from its greater distance from the macrovasculature (Haast et al., 2024). Our findings further revealed that CA1 not only had the lowest CBF but also the longest ATT among hippocampal subfields. The hippocampus relies on a complex vascular network to meet its metabolic demands, with CA1 primarily supplied by ventral arteries, unlike other subfields receiving multiple arterial inputs (Henri et al., 2013; Li et al., 2024). This unique vascular supply and the lengthy course of CA1′s arterial branches help explain its selective vulnerability to anoxia (Henri et al., 2013). Studies show that transient forebrain ischemia selectively induces delayed neuronal death in CA1 pyramidal neurons while sparing other hippocampal and cortical regions (Ouyang et al., 2007; 2013). This vascular dysfunction, combined with neuronal loss, creates a vicious cycle in which reduced blood flow exacerbates neuronal damage, driving CA1 atrophy.

### 4.3. ATT as a sensitive biomarker for hippocampus aging

Age-related hippocampal atrophy was observed across all subfields, with ATT showing greater sensitivity to aging and stronger correlations with atrophy than CBF. Prolonged ATT in gray matter with age has been reported in a few studies using multi-delay ASL (Juttukonda et al., 2021; Liu et al., 2012), aligning with histological and imaging studies showing increased vessel tortuosity in carotid arteries and arterioles with aging (Sun et al., 2022; 2024). Beyond tortuosity, vascular rarefaction and arteriole wall stiffening further prolong blood transit time (Riddle et al., 2003; Sonntag et al., 1997). As a measure of blood travel time from major arteries to microvascular perfusion sites (Damestani et al., 2023), ATT is more sensitive than CBF to subtle vascular changes, such as increased resistance, altered microvascular structure, or impaired flow dynamics, which may precede detectable flow deficits. Prolonged ATT is commonly observed in arterial steno-occlusive disease and AD (Hafdi et al., 2022; Sun et al., 2022). While CBF can partially compensate for reduced blood flow through the local arterial network, ATT may remain largely unaffected by these compensatory mechanisms, making it a valuable imaging marker of structural vascular changes.

The strong correlation between ATT and hippocampal atrophy highlights its potential clinical utility in detecting early vascular impairments before significant CBF reductions occur. Prolonged ATT may contribute to degenerative changes in the hippocampus by exacerbating tissue hypoxia and oxidative stress, leading to neuronal apoptosis and tissue loss (Zhu et al., 2022). Furthermore, impaired neurovascular coupling in aging reduces the hippocampal vasculature’s ability to meet neuronal metabolic demands, compounding the effects of prolonged ATT (Tarantini et al., 2017). Therefore, monitoring ATT might aid in early cerebrovascular assessments, inform targeted vascular interventions, and improve age-related neurodegenerative outcomes.

### 4.4. Limitations and future directions

Several limitations of the study should be acknowledged. First, the ASL images had an isotropic resolution of 2.5 mm, which, although higher than the typical 4 mm isotropic resolution in most 3T studies, may still introduce partial volume effects (PVE), particularly in small or complex regions like hippocampal subfields. To mitigate this, we employed the BASIL toolkit with partial volume correction to reduce PVE. Notably, our finding of the lowest perfusion in CA1 aligns with previous high-resolution (1.5 mm isotropic) ASL studies (Haast et al., 2024), reinforcing the reliability of our results. Future improvements could involve implementing higher-resolution ASL acquisition protocols or integrating super-resolution reconstruction techniques to enhance spatial precision. The second limitation is the lack of blood hormone level measurements. While hormone levels were not directly assessed, we assessed structural and hemodynamic changes in the hypothalamus, which regulates hormone secretion through the HPG axis. These changes may serve as indirect indicators of age-related hormonal variations. Thirdly, after adjusting for age, the observed sex-specific associations between hippocampal subfield metrics and cognition were no longer significant, which may weaken the interpretation of sex-specific structural or hemodynamic coupling with cognition. However, this pattern may reflect a true dependency on aging in general. Despite these limitations, this study was the first attempt that primarily focused on the influence of aging and sex on both hypothalamic and hippocampal subfield structure and hemodynamics, as well as their association with cognitive function. These findings will provide a key reference for identifying abnormal aging processes in the future.

## 5. Conclusion

Our study revealed the relationship between hippocampal structure and vascular function, uncovering sex- and subfield-specific aging trajectories. Menopause-related changes in hypothalamic structure and hemodynamics may contribute to the observed sex differences in hippocampal aging. This study provides a normative reference for abnormal aging processes such as AD and AD-related dementia.

## Supplementary Material

Supp 1

Supplementary materials

Supplementary material associated with this article can be found, in the online version, at doi:10.1016/j.neuroimage.2025.121343.

## Figures and Tables

**Table 1:** Demographic and imaging information of participants.

Variates	Female (*n* = 358)							Male (*n* = 292)						
	36–45y (*n* = 76)	45–55y (*n* = 81)	55–90y (*n* = 201)	*P_1_*	*P_2_*	*P_3_*	Partial η^2^	36–45y (*n* = 58)	45–55y (*n* = 59)	55–90y (*n* = 175)	*P_1_*	*P_2_*	*P_3_*	Partial η^2^

**Clinical data**														
Age, years	40.414 ± 2.421	49.823 ± 2.837	69.890 ± 9.933	**<0.001**	**<0.001**	**<0.001**	0.732	40.066 ± 2.489	50.291 ± 2.893	71.042 ± 9.731	**<0.001**	**<0.001**	**<0.001**	0.742
Total Cognition Composite Score	109.833 ± 10.093	107.813 ± 11.000	103.201 ± 9.892	0.347	**0.003**	**<0.001**	0.074	109.082 ± 10.741	106.792 ± 11.642	103.746 ± 9.060	0.378	0.118	**0.003**	0.047
**Imaging data**														
Volume/eTIV × 100, %														
Hypothalamus	0.057 ± 0.008	0.058 ± 0.008	0.053 ± 0.007	0.768	**<0.001**	**<0.001**	0.091	0.055 ± 0.004	0.054 ± 0.006	0.050 ± 0.006	0.595	**<0.001**	**<0.001**	0.175
Subiculum	0.069 ± 0.010	0.068 ± 0.011	0.061 ± 0.010	0.697	**<0.001**	**<0.001**	0.126	0.064 ± 0.008	0.063 ± 0.007	0.056 ± 0.008	0.629	**<0.001**	**<0.001**	0.169
CA1	0.136 ± 0.019	0.138 ± 0.019	0.121 ± 0.019	0.576	**<0.001**	**<0.001**	0.165	0.126 ± 0.012	0.127 ± 0.013	0.111 ± 0.017	0.766	**<0.001**	**<0.001**	0.201
CA2	0.018 ± 0.003	0.019 ± 0.003	0.017 ± 0.002	0.064	**<0.001**	**0.032**	0.068	0.017 ± 0.002	0.017 ± 0.002	0.016 ± 0.002	0.696	**0.001**	**0.011**	0.053
CA3	0.047 ± 0.007	0.047 ± 0.006	0.044 ± 0.006	0.624	**<0.001**	**0.005**	0.053	0.043 ± 0.004	0.044 ± 0.004	0.042 ± 0.005	0.707	**0.003**	**0.021**	0.045
CA4	0.025 ± 0.005	0.024 ± 0.005	0.022 ± 0.004	0.785	**<0.001**	**<0.001**	0.087	0.023 ± 0.004	0.022 ± 0.004	0.019 ± 0.004	0.766	**<0.001**	**<0.001**	0.128
DG	0.021 ± 0.003	0.021 ± 0.003	0.019 ± 0.003	0.735	**<0.001**	**<0.001**	0.122	0.019 ± 0.002	0.019 ± 0.002	0.017 ± 0.003	0.904	**<0.001**	**<0.001**	0.150
eTIV	1395,376.007 ± 173,650.005	1385,574.786 ± 164,772.477	1448,505.062 ± 147,867.891	0.766	**0.006**	**0.025**	0.033	1613,771.427 ± 127,491.536	1620,167.148 ± 152,350.699	1671,928.513 ± 151,853.151	0.844	**0.038**	**0.019**	0.033
ATT, seconds														
Hypothalamus	1.332 ± 0.095	1.311 ± 0.096	1.371 ± 0.094	0.257	**<0.001**	**0.006**	0.068	1.371 ± 0.081	1.363 ± 0.085	1.379 ± 0.084	0.697	0.280	0.624	0.007
Subiculum	1.177 ± 0.102	1.200 ± 0.089	1.260 ± 0.099	0.214	**<0.001**	**<0.001**	0.123	1.243 ± 0.085	1.257 ± 0.077	1.320 ± 0.082	0.530	**<0.001**	**<0.001**	0.154
CA1	1.292 ± 0.093	1.305 ± 0.099	1.379 ± 0.092	0.530	**<0.001**	**<0.001**	0.157	1.365 ± 0.082	1.366 ± 0.067	1.420 ± 0.073	0.929	**<0.001**	**<0.001**	0.117
CA2	1.242 ± 0.108	1.260 ± 0.103	1.356 ± 0.123	0.466	**<0.001**	**<0.001**	0.172	1.314 ± 0.112	1.330 ± 0.103	1.403 ± 0.098	0.536	**<0.001**	**<0.001**	0.134
CA3	1.188 ± 0.106	1.224 ± 0.107	1.303 ± 0.119	0.083	**<0.001**	**<0.001**	0.160	1.262 ± 0.102	1.290 ± 0.101	1.361 ± 0.093	0.179	**<0.001**	**<0.001**	0.164
CA4	1.133 ± 0.120	1.163 ± 0.126	1.252 ± 0.131	0.221	**<0.001**	**<0.001**	0.146	1.226 ± 0.134	1.237 ± 0.108	1.341 ± 0.122	0.712	**<0.001**	**<0.001**	0.164
DG	1.174 ± 0.109	1.198 ± 0.122	1.300 ± 0.136	0.351	**<0.001**	**<0.001**	0.167	1.271 ± 0.133	1.288 ± 0.105	1.380 ± 0.119	0.576	**<0.001**	**<0.001**	0.148
CBF, ml/100 g/min														
Hypothalamus	49.132 ± 9.315	50.632 ± 15.315	47.214 ± 12.223	0.586	0.067	0.373	0.013	41.750 ± 11.022	41.171 ± 8.447	42.842 ± 13.178	0.828	0.504	0.662	0.003
Subiculum	52.790 ± 10.823	51.628 ± 10.706	51.757 ± 12.982	0.662	0.942	0.650	0.001	44.618 ± 12.296	42.646 ± 8.168	46.323 ± 13.844	0.536	0.092	0.526	0.013
CA1	44.998 ± 8.543	44.991 ± 8.576	40.558 ± 9.499	0.996	**0.001**	**0.001**	0.056	37.535 ± 9.115	36.138 ± 7.221	33.944 ± 9.248	0.536	0.165	**0.016**	0.028
CA2	62.937 ± 13.221	62.282 ± 14.570	54.671 ± 13.851	0.813	**<0.001**	**<0.001**	0.075	52.104 ± 13.613	49.244 ± 10.013	44.545 ± 12.880	0.330	**0.025**	**<0.001**	0.060
CA3	60.195 ± 13.462	61.272 ± 12.977	54.296 ± 12.888	0.703	**<0.001**	**0.002**	0.058	48.938 ± 12.277	48.098 ± 9.559	44.971 ± 11.772	0.766	0.118	**0.042**	0.023
CA4	54.785 ± 10.429	55.973 ± 11.241	51.904 ± 12.446	0.650	**0.018**	0.118	0.022	45.366 ± 10.862	45.654 ± 8.784	44.727 ± 12.304	0.911	0.697	0.770	0.001
DG	55.103 ± 10.056	55.591 ± 10.415	51.933 ± 12.150	0.828	**0.028**	**0.048**	0.025	46.147 ± 10.649	45.403 ± 8.903	44.026 ± 12.092	0.774	0.555	0.328	0.006

Values are expressed as mean ± standard deviation. *P_1_*: difference between 36–45 and 45–55 age group; *P_2_*: difference between 45–55 and 55–90 age group; *P_3_*: difference between 36–45 and 55–90 age group. Statistically significant *p*-values (< 0.05) after false discovery rate (FDR) correction are shown in bold. Partial η^2^ represents the effect size, indicating the proportion of variance in the dependent variable explained by an independent variable, controlling for other variables. Partial η^2^ values of approximately 0.01, 0.06, and 0.14 are interpreted as indicating small, medium, and large effect sizes, respectively. ATT: Arterial transit time; CBF: Cerebral blood flow; DG: Dentate gyrus; eTIV: Estimated total intracranial volume.

## Data Availability

Data from the Human Connectome Project–Aging are publicly available through the National Institute of Mental Health Data Archive (NDA) [https://nda.nih.gov/]. Access requires submission of a Data Use Agreement and a Data Use Certification via the NDA platform. Hippocampal subfield segmentation was performed using the publicly available HippUnfold software (v1.2.0) developed by Khan et al. (2022), available at https://doi.org/10.5281/zenodo.7063098.

## Data availability

Data from the Human Connectome Project-Aging are publicly available through the National Institute of Mental Health Data Archive.
