# Supplementary material for: Hippocampal subfields in aging: Sex-specific trajectories in structure and hemodynamics
Source: Neuroimage. Author manuscript; Available in PMC 2026 Apr 7. (PMC13056313; doi:10.1016/j.neuroimage.2025.121343)
Supplement: Supp 1 [file NIHMS2153844-supplement-Supp_1.docx]

**Supplementary Material 1. Quadratic Models for Age-Related Volume Changes in Hippocampal Subfields**

The following equations represent the fitted quadratic regression models describing the relationships between age and hippocampal subfield volumes for females and males. These models were used to characterize nonlinear volumetric trajectories across the adult lifespan.

**For females:**

$$Subiculum volume=0.057+6.323\times{10}^{-4}\times Age-8.063\times{10}^{-6}\times Age^{2}$$

$$CA1 volume=0.105+1.581\times{10}^{-3}\times Age-1.896\times{10}^{-5}\times Age^{2}$$

$$CA2 volume=0.016+1.173\times{10}^{-4}\times Age-1.331\times{10}^{-6}\times Age^{2}$$

$$CA3 volume=0.040+3.114\times{10}^{-4}\times Age-3.550\times{10}^{-6}\times Age^{2}$$

$$CA4 volume=0.022+1.637\times{10}^{-4}\times Age-2.283\times{10}^{-6}\times Age^{2}$$

$$DG volume=0.016+2.321\times{10}^{-4}\times Age-2.728\times{10}^{-6}\times Age^{2}$$

**For males:**

$$Subiculum volume=0.052+5.897\times{10}^{-4}\times Age-7.296\times{10}^{-6}\times Age^{2}$$

$$CA1 volume=0.100+1.323\times{10}^{-3}\times Age-1.605\times{10}^{-5}\times Age^{2}$$

$$CA3 volume=0.033+4.492\times{10}^{-4}\times Age-4.516\times{10}^{-6}\times Age^{2}$$

$$CA4 volume=0.020 + 1.504\times{10}^{-4}\times Age-2.263\times{10}^{-6}\times Age^{2}$$

$$DG volume=0.014 + 2.279\times{10}^{-4}\times Age-2.681\times{10}^{-6}\times Age^{2}$$

**Supplementary Material 2.** **Age × Sex Interaction Effect**

**Table S1.** Linear regression model of ATT and CBF in each hippocampal subfield.

|  | **Standard β** | | ***P* value** | | **β 95% CI** | | **Adjusted R²** | |
| --- | --- | --- | --- | --- | --- | --- | --- | --- |
|  | ATT | CBF | ATT | CBF | ATT | CBF | ATT | CBF |
| **Subiculum** | | | | | | | | |
| Age | 0.441 | -0.007 | <0.001 | 0.885 | [0.002, 0.004] | [-0.093, 0.080] | 0.255 | 0.071 |
| Sex | 0.453 | -0.518 | 0.001 | 0.001 | [0.036, 0.149] | [-21.106, -5.396] |  |  |
| Age×sex | -0.177 | 0.270 | 0.223 | 0.096 | [-0.001, 0.0003] | [-0.019, 0.235] |  |  |
| **CA1** | | | | | | | | |
| Age | 0.514 | -0.278 | <0.001 | <0.001 | [0.003, 0.004] | [-0.245, -0.121] | 0.263 | 0.197 |
| Sex | 0.661 | -0.494 | <0.001 | 0.001 | [0.075, 0.180] | [-15.459, -4.150] |  |  |
| Age×sex | -0.429 | 0.143 | **0.003** | 0.341 | [-0.002, -0.0004] | [-0.047, 0.136] |  |  |
| **CA2** | | | | | | | | |
| Age | 0.528 | -0.332 | <0.001 | <0.001 | [0.004, 0.005] | [-0.420, -0.237] | 0.263 | 0.229 |
| Sex | 0.582 | -0.488 | <0.001 | 0.001 | [0.076, 0.211] | [-22.811, -6.196] |  |  |
| Age×sex | -0.384 | 0.138 | **0.008** | 0.351 | [-0.003, -0.0003] | [-0.071, 0.198] |  |  |
| **CA3** | | | | | | | | |
| Age | 0.521 | -0.256 | <0.001 | <0.001 | [0.003, 0.005] | [-0.320, -0.147] | 0.292 | 0.199 |
| Sex | 0.483 | -0.531 | <0.001 | <0.001 | [0.052, 0.181] | [-22.411, -6.752] |  |  |
| Age×sex | -0.250 | 0.162 | 0.077 | 0.282 | [-0.002, 0.0001] | [-0.057, 0.196] |  |  |
| **CA4** | | | | | | | | |
| Age | 0.446 | -0.142 | <0.001 | 0.005 | [0.003, 0.005] | [-0.199, -0.036] | 0.285 | 0.122 |
| Sex | 0.347 | -0.502 | 0.012 | 0.001 | [0.022, 0.177] | [-19.888, -5.069] |  |  |
| Age×sex | -0.057 | 0.181 | 0.690 | 0.249 | [-0.002, 0.001] | [-0.050, 0.190] |  |  |
| **DG** | | | | | | | | |
| Age | 0.487 | -0.158 | <0.001 | 0.002 | [0.004, 0.005] | [-0.207, -0.049] | 0.296 | 0.143 |
| Sex | 0.468 | -0.435 | 0.001 | 0.004 | [0.057, 0.209] | [-17.812, -3.429] |  |  |
| Age×sex | -0.189 | 0.092 | 0.180 | 0.556 | [-0.002, 0.0004] | [-0.081, 0.151] |  |  |

Statistically significant age × sex interactions (*p* < 0.05) are highlighted in bold. CI: Confidence interval; ATT: Arterial transit time; CBF: Cerebral blood flow; DG: Dentate gyrus.
